# Outcomes and antibiotic use in patients with coronavirus disease 2019 (COVID-19) admitted to an intensive care unit

**DOI:** 10.1017/ash.2021.248

**Published:** 2022-01-17

**Authors:** Megan M. Petteys, Leigh Ann Medaris, Julie E. Williamson, Rohit S. Soman, Travis A. Denmeade, William E. Anderson, Michael K. Leonard, Christopher M. Polk

**Affiliations:** 1 Antimicrobial Support Network, Atrium Health, Charlotte, North Carolina; 2 Division of Infectious Diseases, Atrium Health, Charlotte, North Carolina; 3 Infectious Disease, Wake Forest Baptist Medical Center, Winston-Salem, North Carolina; 4 Center for Outcomes Research and Evaluation, Atrium Health, Charlotte, North Carolina

## Abstract

Antibiotic overuse is high in patients hospitalized with coronavirus disease 2019 (COVID-19) despite a low documented prevalence of bacterial infections in many studies. In this study evaluating 65 COVID-19 patients in the intensive care unit, empiric broad-spectrum antibiotics were often overutilized with an inertia to de-escalate despite negative culture results.

Nearly three-quarters of patients with coronavirus disease 2019 (COVID-19) receive antibiotics even though the prevalence of bacterial coinfections is low (ie, <10% in many studies^1–4^) and ranges from 8% to 22% in studies of patients in an intensive care unit (ICU).^3–5^ Broad-spectrum antibiotics (BSAs), such as piperacillin–tazobactam, are commonly prescribed for a mean duration of 5 to 7 days.^6^ However, antibiotics appear unlikely to improve outcomes in hospitalized COVID-19 patients.^7^ Antibiotic overuse in COVID-19 patients may be associated with unintended consequences including adverse events and the development of antimicrobial resistance. The purpose of this study was to examine outcomes and antibiotic use in ICU COVID-19 patients treated with an empiric antibiotic course compared to patients with documented bacterial infection and those whom antibiotics were stopped ≤72 hours given negative cultures.

## Methods

This retrospective, multicenter cohort study evaluated adults with COVID-19 started on a course of antibiotics in an ICU at 1 of 10 hospitals within a large healthcare system between September and December 2020. Patients were considered for inclusion for the first episode of antibiotics initiated following a positive severe acute respiratory coronavirus virus 2 (SARS-CoV-2) result (the index regimen) and if blood and/or respiratory cultures were obtained ≤24 hours of antibiotic initiation. We applied the following exclusion criteria: death ≤72 hours of antibiotic initiation, antibiotics initiated prior to a positive SARS-CoV-2 result, transfer from an outside hospital, antibiotics initiated while in a non-ICU unit, or patient already on antibiotics for an alternate indication (eg, prophylaxis or suppression or antibiotics continued upon admission). Patients were still considered for inclusion if empiric antibiotics were ordered and then discontinued upon confirmation of SARS-CoV-2 infection, as long as they met the criteria above for the index regimen and culture obtainment. Prior antibiotic exposure was defined as receipt of intravenous antibiotics for ≥48 hours within the last 90 days. Multiple antibiotic courses following the index regimen were assessed but were not considered unique events. Eligible patients were categorized into 1 of 3 groups: (1) culture-negative, antibiotics discontinued ≤72 hours, (2) culture-negative, antibiotics continued >72 hours, or (3) culture-positive.

Following institutional review board approval, patients were identified using electronic surveillance software and were followed until hospital discharge or in-hospital death. Electronic medical record review and Research Electronic Data Capture (REDCap) were used to collect and organize the following data: comorbidities with a high risk of progressing to severe COVID-19 illness (identified by study investigator during record review rather than ICD-10 code), supplemental oxygen requirement at antibiotic initiation, COVID-19-specific therapy, microbiological culture information, and antibiotic use.

The primary outcome was clinical success, defined as being discharged alive or >2-point decrease in the World Health Organization Clinical Progression Scale score from day of antibiotic initiation to day 30.^8^ Secondary outcomes included time from antibiotic discontinuation until restart, in-hospital mortality, and time from antibiotic initiation to ICU discharge and hospital discharge or death. Adverse events were also assessed, including acute kidney injury, development of multidrug resistance, antibiotic-related rash, and *Clostridioides difficile* infection. Acute kidney injury as defined by the Acute Kidney Injury Network criteria was assessed following 24 hours of the initial regimen and up to 48 hours after discontinuation.^9^ Multidrug-resistant organisms included methicillin-resistant *Staphylococcus aureus* (MRSA), vancomycin-resistant *Enterococcus*, or Gram-negative bacteria resistant to 1 or more classes of antimicrobial agents per the Centers for Disease Control and Prevention definition.^10^

### Statistical analysis

The 3 groups were compared on baseline characteristics, antibiotic use, outcomes, and adverse events using the χ^2^ test or the Fisher exact test for nominal variables, the Kruskal-Wallis test for ordinal variables and time variables, and one-way analysis of variance for other interval variables. A 2-tailed *P* value of <.05 was considered statistically significant. No adjustments were made for multiple testing. All analyses were conducted using SAS Enterprise Guide version 7.1 software (SAS Institute, Cary, NC).

## Results

During the 4-month study period, of 296 ICU COVID-19 patients screened, only 65 (22%) met inclusion criteria. The most common exclusions were antibiotic initiation prior to confirmed positive SARS-CoV-2 result (n = 84), no culture obtainment (n = 44), and non-ICU admission upon antibiotic initiation (n = 37). Of the 65 included patients, 23 (35%) had culture-positive infection; 32 (49%) were culture-negative and had antibiotics continued >72 hours; and 10 (15%) were culture-negative and had antibiotics discontinued ≤72 hours of initiation.

**Fig. 1.**
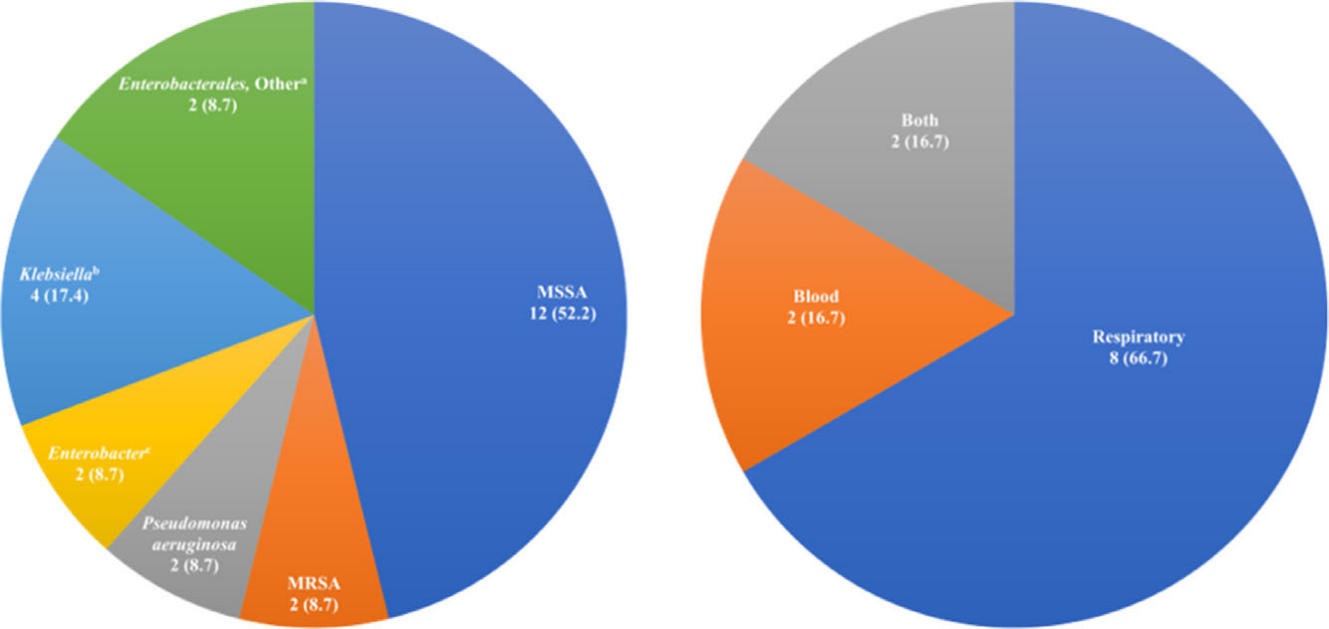
Most common bacterial pathogens isolated (*left*), no. (%, N = 23) and MSSA by culture type (*right*), no. (%, N = 12). Note. MRSA, methicillin-resistant *Staphylococcus aureus;* MSSA, methicillin-susceptible *S. aureus.* ^a^*Citrobacter koseri*, *Escherichia coli*, *Proteus mirabilis*, *Serratia marcescens*. ^b^*Klebsiella pneumoniae*, *Klebsiella variicola.* ^c^*Enterobacter aerogenes*, *Enterobacter cloacae.*

Table 1 includes results on baseline characteristics, antibiotic use, outcomes, and adverse events. We detected no significant differences between the groups in terms of baseline demographics, comorbidities, oxygen requirements, or COVID-19 therapies. BSAs with anti-MRSA and anti-pseudomonal activity were commonly prescribed as the initial regimen in all 3 groups. Relative to the culture-negative groups, anti-MRSA agents were less frequently prescribed in the culture-positive group, where cefazolin was utilized more often. This may be related to the preponderance of methicillin-susceptible *Staphylococcus aureus* (MSSA) isolated primarily from respiratory cultures in this group (Fig. 1). Median treatment duration for MSSA was 10.5 days if isolated in respiratory cultures and 11.5 days if isolated in blood cultures (or both). Overall, median duration of initial therapy was 9 days. De-escalation occurred more often in the culture-positive group, although these patients received longer antibiotic courses compared to both culture-negative groups.

**Table 1.** Baseline Characteristics, Antibiotic Use, Outcomes, and Adverse Events in ICU Patients with COVID-19 (N = 65)

Baseline Characteristics	Culture-Negative, Antibiotics ≤72 h (n = 10)	Culture-Negative, Antibiotics >72 h (n = 32)	Culture- Positive (n = 23)	*P* Value
Age, mean (SD)	66.1 (13.5)	66 (11.9)	62.4 (13.4)	.56
Sex, no. (% male)	7 (70)	19 (59.4)	15 (65.2)	.80
**Race, no. (%)**				.36
White	8 (80)	16 (50)	15 (65.2)	
Black or African American	2 (20)	11 (34.4)	5 (21.7)	
Ethnicity, no. (% Hispanic or Latino)	1 (10)	4 (12.5)	3 (13)	1.0
BMI (kg/m^2^), mean (SD)	32.7 (7.9)	34 (12.3)	32.5 (6.7)	.86
Charlson comorbidity index, mean (SD)	3.4 (2.2)	4.3 (3.1)	3.4 (2)	.43
APACHE II score, mean (SD)	21.4 (13.6)	21.4 (8.3)	20.2 (7.7)	.88
Clinical progression scale score at antibiotic initiation,median (IQR)	6.5 (6–8)	7.5 (6–8)	8 (7–9)	.22
**Comorbidities, no. (%)**				
Cardiovascular disease	6 (60)	24 (75)	16 (69.6)	.65
Smoking	4 (40)	8 (25)	11 (47.8)	.21
Diabetes	3 (30)	17 (53.1)	11 (47.8)	.44
Chronic lung disease	3 (30)	5 (15.6)	6 (26.1)	.51
CKD/ESRD	2 (20)	7 (21.9)	2 (8.7)	.49
Active malignancy	0	3 (9.4)	2 (8.7)	1.0
Liver disease	1 (10)	2 (6.3)	1 (4.3)	.81
Cerebrovascular disease	0	3 (9.4)	1 (4.3)	.81
History of MDRO, no. (%)	0	0	0	1.0
History of prior antibiotic exposure, no. (%)	3 (30)	6 (18.8)	9 (39.1)	.25
**Supplemental O**_**2**_ **requirement at antibiotic initiation, no. (%)**				.18
Invasive mechanical ventilation	4 (40)	20 (62.5)	18 (78.3)	
Noninvasive ventilation or high-flow device	3 (30)	6 (18.8)	4 (17.4)	
Nasal cannula				
<6 L/min	2 (20)	3 (9.4)	1 (4.3)	
≥6 L/min	0	3 (9.4)	0	
ECMO	1 (10)	0	0	
**COVID-19 therapy, no. (%)**				
Remdesivir	10 (100)	27 (84.4)	23 (100)	.07
Dexamethasone	10 (100)	31 (96.9)	22 (95.7)	1.0
Convalescent plasma	6 (60)	16 (50)	11 (47.8)	.81
**Antibiotic use, no. (%) or median (IQR)**				
**Initial antibiotic regimen** ^a^.^b^				
Anti-MRSA	8 (80)	21 (65.6)	11 (47.8)	.17
Anti-pseudomonal	8 (80)	27 (84.4)	14 (60.9)	.13
Ceftriaxone	1 (10)	5 (15.6)	3 (13)	1
Cefazolin	0	0	4 (17.4)	.02
Initial regimen de-escalated	2 (20)	19 (59.4)	17 (73.9)	.02
Initial regimen continued (not de-escalated/escalated)	8 (80)	10 (31.3)	3 (13)	<.01
Duration of therapy, initial regimen, d	2 (1–3)	5 (4–6)	9 (6–13)	<.01
Antibiotics restarted ≥24 h after discontinuation	4 (40)	15 (46.9)	5 (21.7)	.16
Duration of therapy, total antibiotics, d	3 (2–6)	6 (5–14)	10 (7–17)	<.01
Time since ICU admission to antibiotic start, d	4 (0–10)	6.5 (2.5–10)	8 (6–12)	.06
Time since positive SARS-CoV-2 test to antibiotic start, d	9.5 (4–14)	10 (7.5–12.5)	12 (10–18)	.09
**Clinical outcomes, no (%) or median (IQR)**				
Clinical success	4 (40)	10 (31.3)	9 (39.1)	.79
In-hospital mortality	6 (60)	22 (68.8)	14 (60.9)	.79
Time from antibiotic discontinuation to re-start, d	4.5 (2–7)	4 (2–8)	6 (4–6)	.68
Time to ICU discharge from antibiotic start, d	8.5 (4–15)	11.5 (5.5–21)	11 (6–15)	.57
Time to hospital discharge or death from antibiotic start, d	12.5 (4–16)	18.5 (6.5–25.5)	13 (8–34)	.29
**Adverse events, no (%)**				
Total patients	2 (20)	13 (40.6)	7 (30.4)	.44
AKI	1 (10)	8 (25)	4 (17.4)	.71
MDRO	0	8 (25)	2 (8.7)	.14
Antibiotic-related rash	1 (10)	0	1 (4.3)	.13
Drug fever	0	1 (3.1)	0	1
*Clostridioides difficile* infection	0	0	0	1

Note. AKI, acute kidney injury; APACHE, Acute Physiology and Chronic Health Evaluation; BMI, body mass index; CKD, chronic kidney disease; d, days; ESRD, end-stage renal disease; ECMO, extracorporeal membrane oxygenation; h, hours; ICU, intensive care unit; IQR, interquartile range; MRSA, methicillin-resistant *Staphylococcus aureus;* MDRO, multidrug-resistant organism; SD, standard deviation.

a Categories of initial antibiotics administered in study. Anti-MRSA: vancomycin, linezolid, ceftaroline (no patient received daptomycin for initial therapy). Anti-pseudomonal: cefepime, piperacillin-tazobactam, aztreonam.

b No broad-spectrum antibiotics targeting Gram-negative MDRO were given for initial therapy (carbapenems, ceftolozane-tazobactam, ceftazidime-avibactam, meropenem-vaborbactam, imipenem-cilastatin-relebactam, cefiderocol).

Outcomes of clinical success, in-hospital mortality, time to restart of antibiotics, and time to ICU and hospital discharge or death did not differ between the groups. Discontinuing antibiotics ≤72 hours if culture-negative did not impact clinical outcomes compared with groups treated with longer antibiotic courses with or without positive cultures. The highest rates of adverse events occurred among the 40.6% of patients who were culture-negative and had antibiotics continued >72 hours. However, these events did not reach statistical significance.

## Discussion

Consistent with prior published literature, ICU COVID-19 patients were often treated with empiric BSAs despite low rates of corroborating culture-positive infections. The observed rate of 35% of COVID-19 patients with positive cultures in our study was higher than the rates described in other cohorts. However, in examining a variety of clinical outcomes, we identified no benefit for continuing antibiotics >72 hours in ICU COVID-19 patients with negative cultures. Instead, there was suggestion of harm with more adverse events in our cohort of patients treated >72 hours with negative cultures.

Also consistent with prior data, BSAs with anti-MRSA and anti-pseudomonal activity were the most frequently prescribed despite low rates of cultures with these organisms. Given that MSSA accounted for nearly half the positive cultures in our study, opportunities for narrowing antibiotic regimens are evident. Antimicrobial stewardship efforts may also focus on ensuring appropriate treatment durations in this group based on the 10.5-day median duration observed for respiratory cultures.

This study had several limitations. The study design was retrospective, we had a small sample size, and the study was conducted over a relatively short period prior to the emergence of new SARS-CoV-2 variants and the adjunctive use of tocilizumab or baricitinib. However, it was a multicenter study including multiple ICUs of both tertiary-level and rural community hospitals. It also represents real-world experience in treating ICU COVID-19 patients.

In conclusion, significant opportunities exist to limit antibiotic exposure in ICU COVID-19 patients because empiric BSAs do not appear to be associated with benefit and may result in harm. When antibiotics are prescribed, spectrum of activity should be targeted at commonly identified pathogens. We observed high rates of anti-MRSA and anti-pseudomonal antibiotic use despite low rates of cultures with these pathogens.

## Research transparency and reproducibility

The data set is available upon request.
